# The association between moral identity and prosocial behavior among college students: multiple mediating effects of moral sensitivity and moral disengagement

**DOI:** 10.3389/fpsyg.2026.1818710

**Published:** 2026-05-21

**Authors:** Junhe Liao, Hui Yu, Zhiyong Li, Danqi Wang, Mingzheng Wu, Zikan Li

**Affiliations:** 1 School of Education, Huainan Normal University, Huainan, China; 2 Department of Psychology and Behavioral Sciences, Zhejiang University, Hangzhou, China; 3 School of Business, City University of Macau, Macau, China

**Keywords:** college students, moral disengagement, moral identity, moral sensitivity, prosocial behavior

## Abstract

The present study aims to further elucidate the underlying associations through which moral identity is positively related to prosocial behavior among college students by examining the mediating roles of moral sensitivity and moral disengagement. A total of 502 undergraduate students participated in the survey, completing the Moral Identity Scale, the Dispositional Moral Sensitivity Questionnaire, the Moral Disengagement Scale, and the Prosocial Tendencies Measure. The results revealed several key findings. First, both internalized and symbolized moral identity were significantly and positively correlated with moral sensitivity and prosocial behavior. Specifically, internalized moral identity was negatively associated with moral disengagement, while symbolized moral identity showed no significant correlation with moral disengagement. Moral sensitivity was positively associated with both moral disengagement and prosocial behavior, whereas moral disengagement was negatively associated with prosocial behavior. Second, moral sensitivity and moral disengagement played multiple mediating roles in the relationship between internalized moral identity and prosocial behavior, showing three distinct indirect pathways: the independent mediation of moral sensitivity, the independent mediation of moral disengagement, and a chained mediation involving both variables. Third, in the relationship between symbolized moral identity and prosocial behavior, the mediating role of moral sensitivity alone and the chained mediation involving both moral sensitivity and moral disengagement were also supported. Overall, this study provides a comprehensive description of the psychological processes through which moral identity is positively associated with prosocial behavior among college students, with findings consistent with the proposed mediation model. The results offer valuable theoretical insights and practical implications for moral education and behavioral development in higher education settings, though causal interpretations should be made with caution due to the cross-sectional design.

## Introduction

The rapid acceleration of globalization has significantly intensified the diversification of values. The widespread prevalence of technological alienation and materialism has led to a marked weakening of traditional ethical systems. This trend is particularly evident in the educational field, where performance indicators have increasingly overshadowed moral cultivation. In the broader social context, the dominance of competitive logic has eroded altruistic values. Under the tension between the decline of value rationality and the dominance of instrumental rationality in contemporary society, moral education for college students has emerged as a crucial issue in addressing the ethical dilemmas of modernity (Landry, 2022). Prosocial behavior, as a significant external manifestation of an individual's moral level, refers to actions that are consciously taken in social interactions to benefit others or society (Li et al., 2025). Such behavior not only is positively associated with positive interpersonal relationships but also contributes significantly to social harmony and progress (Yang et al., 2016; Wu et al., 2017). Therefore, identifying the key factors that influence college students' prosocial behavior has become a focal point in recent research.

Moral identity is widely recognized as a core psychological mechanism through which moral cognition is translated into moral action (Aquino et al., 2009). Drawing on social cognitive theory, moral identity is defined as the internalization of moral traits such as fairness, compassion, and loyalty into an individual's self-concept (Lee et al., 2014). It functions as a fundamental internal motivator for moral behaviors, including prosocial actions (Yang et al., 2016; Zheng et al., 2022). Extant research has consistently shown that individuals with a higher level of moral identity are more likely to engage in prosocial behavior (Wang et al., 2019; Yang et al., 2018). Meta-analyses have further confirmed a stable and positive association between moral identity and prosocial behavior (Intishar et al., 2024). Specifically, individuals with high moral identity are more willing to participate in volunteer services, charitable donations, and other altruistic behaviors (Wu and Yang, 2018; Winterich et al., 2013; Wang, 2017). These individuals not only donate more but are also more inclined to help strangers. Moreover, they exhibit lower tendencies toward bullying (Reynolds, 2008) and significantly reduced cyberbullying behaviors (Hardy, 2006), demonstrate fewer antisocial behaviors in competitive settings (Liu and Zhang, 2020), and are less likely to seek revenge even when harmed (Li and Hu, 2023). Based on these findings, this study proposes the following hypothesis:

**Hypothesis 1:** Moral identity positively predicts college students' prosocial behavior.

Aquino and colleagues have further differentiated two dimensions of moral identity based on individuals' concerns about self-image: internalization and symbolization (Hertz and Krettenauer, 2016). The internalized dimension of moral identity emphasizes the centrality of moral traits within an individual's self-structure, representing the “private self” (Lee et al., 2014). In contrast, the symbolized dimension of moral identity focuses on the outward display of moral traits to others, aligning with the “public self” (Fang and Huang, 2023). Although both dimensions of moral identity can influence prosocial behavior, prior studies have suggested differences in their predictive power and stability (Gollwitzer et al., 2005). For instance, symbolized moral identity is more susceptible to external is positively associated with, such as social evaluation or ego depletion (Fan, 2013; McMahon et al., 2006). Given these distinctions, this study proposes the following hypothesis:

**Hypothesis 2:** Both internalized and symbolized moral identity predict prosocial behavior, but through different mechanisms.

Previous studies have suggested that moral sensitivity and moral disengagement may serve as facilitators and inhibitors, respectively, in the process by which moral identity affects prosocial behavior (Wang, 2017; McMahon et al., 2006), thus jointly regulating this relationship. Moral sensitivity, which refers to an individual's ability and tendency to recognize moral issues in a given context (Barr et al., 2007), has been found to be positively associated with prosocial behavior and negatively correlated with antisocial behavior (Schmitt et al., 2005). This positive correlation between moral sensitivity and prosocial behavior also holds true in digital contexts (Schmitt et al., 2005). Moreover, the components of moral sensitivity, such as empathy and perspective-taking, have been shown to be positively related to prosocial actions (Yang and Liang, 2022; Bandura, 2002).

Furthermore, empirical research has demonstrated that moral identity positively predicts moral sensitivity (Li et al., 2025). Individuals with a high level of moral identity are more likely to activate moral self-schemas in moral contexts, which in turn enhances their sensitivity to moral issues and is positively associated with prosocial behavior. Given these findings, this study proposes the following hypothesis:

**Hypothesis 3:** Moral sensitivity mediates the relationship between moral identity and prosocial behavior.

On the other hand, moral disengagement is a cognitive strategy that enables individuals to temporarily suspend their moral standards, thereby reducing self-condemnation arising from moral transgressions (Earp, 2022; Hardy et al., 2015). Studies have consistently demonstrated a negative correlation between moral disengagement and prosocial behavior, with higher levels of moral disengagement being associated with fewer prosocial acts (Luo and Pan, 2023; Johnson et al., 2024). Moreover, individuals with a high level of moral identity are less likely to adopt moral disengagement strategies (Lützén and Evertzon, 2010). Based on these findings, this study proposes the following hypothesis:

**Hypothesis 4:** Moral disengagement mediates the relationship between moral identity and prosocial behavior.

Finally, when confronted with the costs associated with moral actions, individuals may develop defensive psychological mechanisms that trigger disengagement strategies to rationalize unethical choices (Barr et al., 2007). Given this understanding, this study proposes the following hypothesis:

**Hypothesis 5:** Moral sensitivity and moral disengagement jointly mediate the relationship between moral identity and prosocial behavior in a sequential (chain) manner.

**Figure 1:**
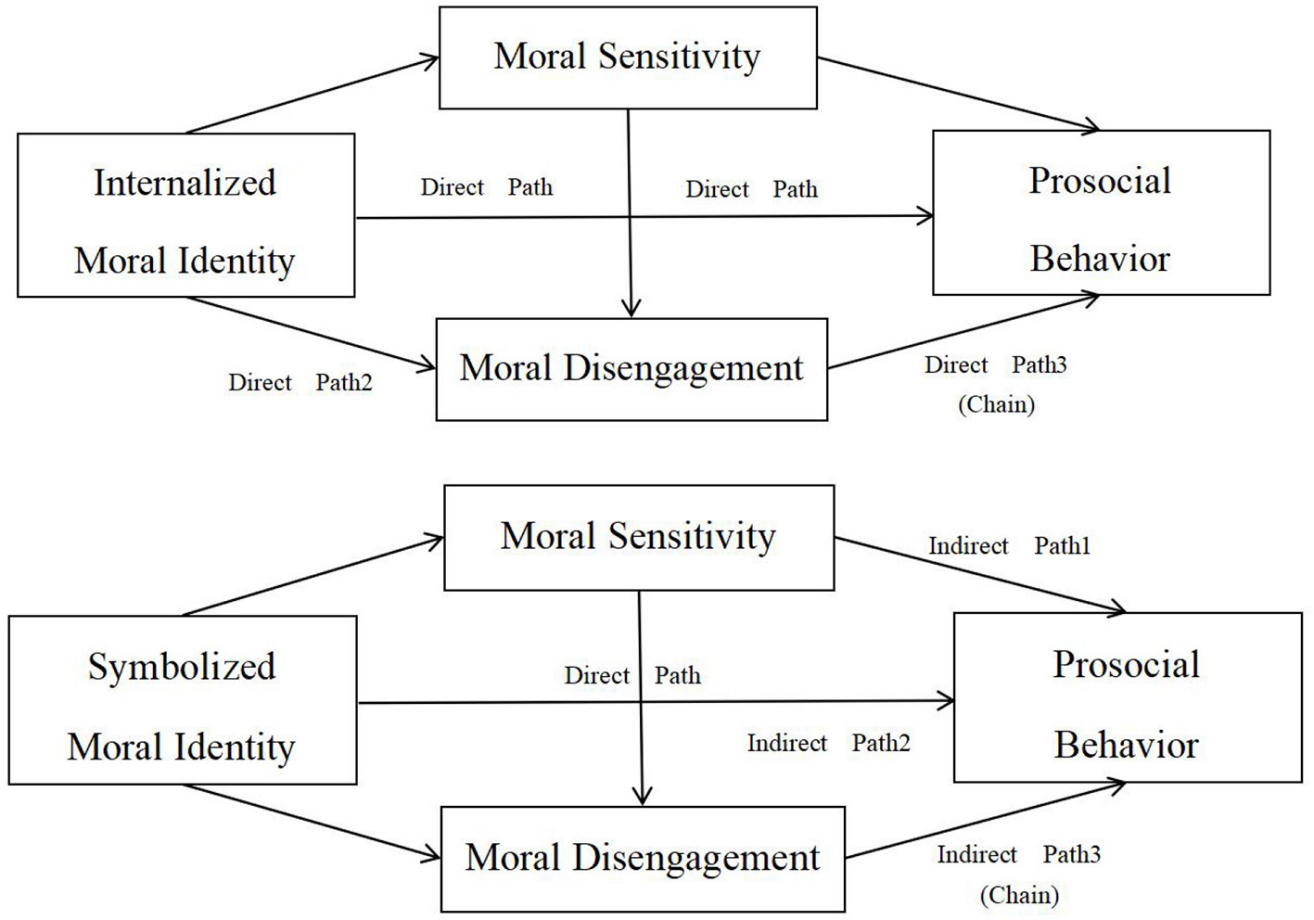
Hypothetical models for internalized and symbolized moral identity.

In summary, this study constructs a multiple mediation model (see Figure 1), with moral identity (both internalized and symbolized dimensions) as the independent variable, prosocial behavior as the dependent variable, and moral sensitivity and moral disengagement as the mediators. The primary objective is to systematically examine the pathways and underlying mechanisms through which moral identity is positively associated with prosocial behavior, thereby offering valuable theoretical insights and empirical support for moral education and behavioral development in higher education settings.

## Methods

### Participants

This study utilized a convenience sampling method to recruit undergraduate students from two universities located in Anhui Province and Shandong Province, China. A total of 560 paper-based questionnaires were distributed, yielding 524 valid responses. In accordance with the recommendations of Luo and Pan (2023), 12 questionnaires with scores exceeding the mean plus two standard deviations on the Social Desirability Scale were excluded to mitigate social desirability bias. This strict exclusion criterion was applied to ensure the high authenticity and quality of the self-reported behavioral tendencies. Additionally, a sensitivity analysis retaining these 12 cases yielded substantially similar results, indicating that the findings are robust. The final sample comprised 502 valid cases, including 126 male and 376 female participants. Among them, 224 were majoring in humanities and 278 in science and engineering. The average age of the participants was 18.93 years (SD = 0.90).

### Measures

#### Moral identity scale (MIS)

In this study, the Moral Identity Scale revised by Wu et al. (Johnson et al., 2024) was employed. This scale consists of 14 items and encompasses two dimensions: internalization and symbolization. Participants were asked to respond using a five-point Likert scale, ranging from 1 (strongly disagree) to 5 (strongly agree), with higher scores indicating a stronger moral identity. In the current study, the Cronbach's alpha coefficients for the internalization and symbolization dimensions were 0.87 and 0.77, respectively, and the validated factor analysis fit index was good, which demonstrate good reliability and validity.

#### Dispositional moral sensitivity questionnaire (DMSQ)

This study employed the Dispositional Moral Sensitivity Questionnaire (DMSQ) developed by Zheng et al. (2022). The questionnaire comprises 28 items that cover five dimensions: empathic guilt, punitive tendency, empathic distress, awareness frequency, and empathic phenomenon. Participants responded using a five-point Likert scale, ranging from 1 (strongly disagree) to 5 (strongly agree), with higher scores indicating greater moral sensitivity. In the current study, the internal consistency coefficient for the overall scale was 0.84, and the validated factor analysis fit index was good, indicating satisfactory reliability.

#### Moral disengagement scale (MDS)

The present study utilized the Chinese version of the Moral Disengagement Scale, which was revised by Ding et al. (2024). This scale comprises 26 items that measure eight factors: moral justification, euphemistic labeling, advantageous comparison, diffusion of responsibility, displacement of responsibility, distortion of consequences, dehumanization, and attribution of blame. Participants responded using a five-point Likert scale, ranging from 1 (strongly disagree) to 5 (strongly agree), with higher scores indicating a stronger tendency toward moral disengagement. In this study, the Cronbach's alpha coefficient for the scale was 0.80, and the validated factor analysis fit index was good, demonstrating good reliability.

#### Prosocial tendencies measure (PTM)

The study employed the Chinese revised version of the Prosocial Tendencies Measure (PTM) for adolescents, developed by Carlo and Randall (2002). This version of the PTM comprises 26 items that assess six dimensions of prosocial behavior: public, anonymous, altruistic, compliant, emotional, and dire prosocial tendencies. Participants were asked to rate each item on a five-point Likert scale, ranging from 1 (not at all like me) to 5 (very much like me), with higher scores indicating stronger prosocial tendencies. In the current study, the Cronbach's alpha coefficient for the full scale was 0.90, and the validated factor analysis fit index was good, indicating excellent reliability.

#### Social desirability scale (SDS)

The short version of the Social Desirability Scale (SDS) developed by Reynolds (1982) was employed in this study. This version of the SDS consists of 13 dichotomous (yes/no) items. Specifically, items 5, 7, 9, 10, and 13 were scored 1 point for a “yes” response, while all other items were scored 1 point for a “no” response. Higher scores on this scale indicate a stronger tendency toward social desirability. In the current study, the internal consistency coefficient of the scale was 0.73, which is within the acceptable range for reliability.

### Statistical methods

The data collected from the questionnaires were entered and organized using Microsoft Excel 2016. Subsequently, statistical analyses were carried out using SPSS version 25.0. The primary analytical methods employed in this study included descriptive statistics, correlation analyses, and multiple mediation analyses.

## Results

### Test for common method bias

To assess whether the data in this study were affected by serious common method bias, Harman's single-factor test was conducted, following the recommendations of Zhou and Long (Podsakoff et al., 2012). The results of the unrotated exploratory factor analysis revealed 25 factors with eigenvalues greater than 1, accounting for a total variance of 62.78%. The first common factor explained only 12.17% of the total variance, which is well below the critical threshold of 40%. These results suggest that common method bias is not a severe issue, though it cannot be entirely eliminated.

### Descriptive statistics and correlation analysis

Pearson product–moment correlation analysis was conducted to examine the relationships among moral identity, moral sensitivity, moral disengagement, and prosocial behavior. The detailed statistical results are presented in Table 1.

**Table 1:** Correlation analysis among moral identity, moral sensitivity, moral disengagement, and prosocial behavior (*N* = 502).

No.	Variable	*M* ± SD	1	2	3	4	5
1	Internalized moral identity	31.17 ± 3.62	1				
2	Symbolized moral identity	24.80 ± 4.08	0.48^***^	1			
3	Moral sensitivity	87.54 ± 12.02	0.14^**^	0.22^***^	1		
4	Moral disengagement	52.01 ± 9.75	−0.10^*^	0.07	0.14^**^	1	
5	Prosocial behavior	94.72 ± 11.54	0.35^***^	0.32^***^	0.29^***^	−0.23^***^	1

^*^p < 0.05;

^**^p < 0.01;

^***^p < 0.001.

As shown in Table 1, internalized moral identity was significantly positively correlated with moral sensitivity and prosocial behavior, and significantly negatively correlated with moral disengagement. Symbolized moral identity also showed significant positive correlations with moral sensitivity and prosocial behavior; however, its correlation with moral disengagement was not statistically significant. Moral sensitivity was significantly positively correlated with both moral disengagement and prosocial behavior, while moral disengagement was significantly negatively correlated with prosocial behavior.

### Multiple mediation analysis of moral sensitivity and moral disengagement

**Figure 2:**
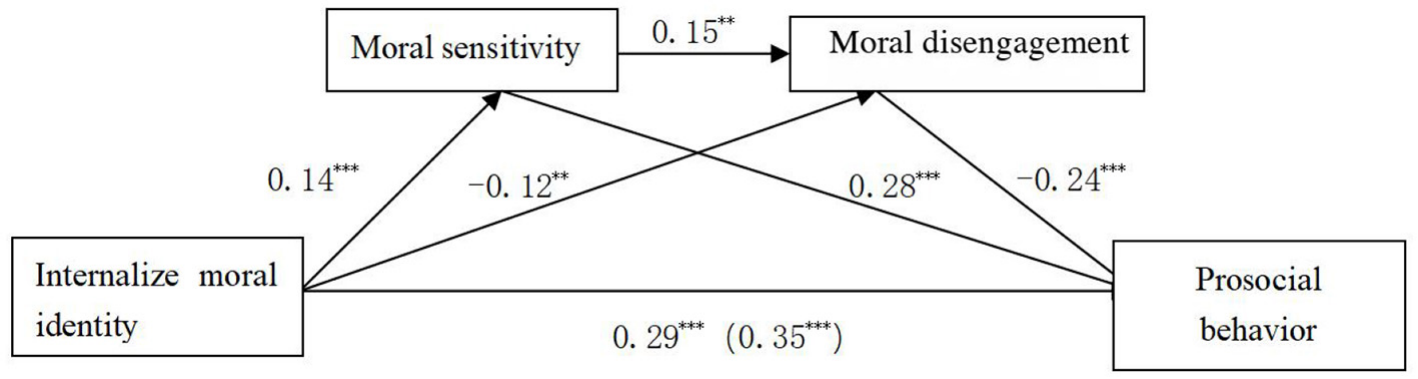
Multiple mediation model M1. *p* < 0.01, ^*^p < 0.001.

To examine the mediating effects of moral sensitivity and moral disengagement in the relationship between moral identity and prosocial behavior, this study employed the PROCESS macro for SPSS developed by Hayes (2012). Internalized and symbolized moral identity were separately entered as independent variables. To rule out potential demographic confounding effects, gender, age, and major were entered into the models as covariates. The bootstrap method with 5,000 resamples was used to compute 95% confidence intervals and construct multiple mediation models (Models M1 and M2). The detailed results are presented in Figures 1, 2, and Tables 2, 3.

**Table 2:** Mediating effects of moral sensitivity and moral disengagement (model M1).

Effect Pathways	Effect value	Proportion of total effect	Boot LLCI	Boot ULCI
Indirect effect 1 (via moral sensitivity)	0.04	0.04/0.35 = 0.11	0.02	0.07
Indirect effect 2 (via moral disengagement)	0.03	0.03/0.35 = 0.09	0.01	0.05
Indirect effect 3 (via both in sequence)	−0.01	−0.01/0.35 = −0.03	−0.01	−0.001
Total indirect effect	0.06	0.06/0.35 = 0.17	0.03	0.10
Direct effect	0.29	0.29/0.35 = 0.83	0.21	0.36
Total effect	0.35			

Boot LLCI, lower limit of 95% bootstrap confidence interval; Boot ULCI, upper limit of 95% bootstrap confidence interval.

**Table 3:** Mediating effects of moral sensitivity and moral disengagement (model M2).

Effect Pathways	Effect Value	Proportion of total effect	Boot LLCI	Boot ULCI
Indirect effect 1 (via moral sensitivity)	0.06	0.06/0.32 = 0.19	0.03	0.07
Indirect effect 2 (via moral disengagement)	−0.01	−0.01/0.32 = −0.03	−0.05	0.01
Indirect effect 3 (via both in sequence)	−0.01	−0.01/0.32 = −0.03	−0.02	−0.002
Total indirect effect	0.04	0.04/0.32 = 0.13	0.001	0.08
Direct effect	0.28	0.28/0.32 = 0.87	0.20	0.36
Total effect	0.32			

Boot LLCI, lower limit of 95% bootstrap confidence interval; Boot ULCI, upper limit of 95% bootstrap confidence interval.

As illustrated in Figure 1 and Table 2, internalized moral identity exerted a significant direct effect on prosocial behavior, with a direct effect value of 0.29, accounting for 83% of the total effect. Additionally, internalized moral identity had a significant indirect effect on prosocial behavior, with an indirect effect value of 0.06, representing 17% of the total effect. This indirect effect was mediated through three distinct pathways:

(1) Internalized Moral Identity → Moral Sensitivity → Prosocial Behavior: The effect size was 0.04, accounting for 11% of the total effect.
(2) Internalized Moral Identity → Moral Disengagement → Prosocial Behavior: The effect size was 0.03, accounting for 9% of the total effect.
(3) Internalized Moral Identity → Moral Sensitivity → Moral Disengagement → Prosocial Behavior: The effect size was −0.01, accounting for −3% of the total effect.

**Figure 3:**
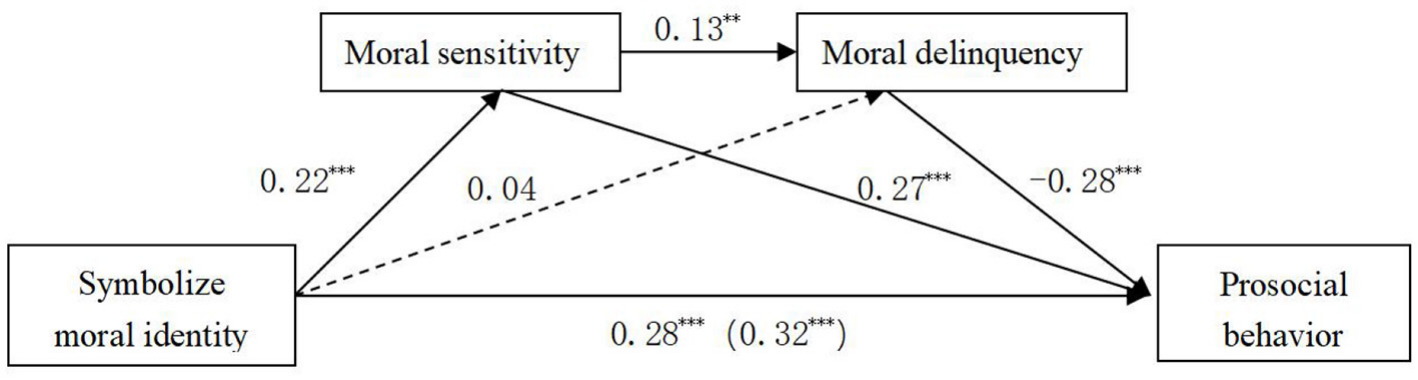
Multiple mediation model M2. *p* < 0.01, ^*^p < 0.001.

As depicted in Figure 3 and Table 3, symbolized moral identity had a significant direct effect on prosocial behavior, with a direct effect value of 0.28, accounting for 87% of the total effect. The indirect effect of symbolized moral identity on prosocial behavior was also significant, with an effect value of 0.04, representing 13% of the total effect. This indirect effect was mediated through two distinct pathways:

(1) Symbolized Moral Identity → Moral Sensitivity → Prosocial Behavior: The effect size was 0.06, accounting for 19% of the total effect.
(2) Symbolized Moral Identity → Moral Sensitivity → Moral Disengagement → Prosocial Behavior: The effect size was −0.01, accounting for −3% of the total effect.

## Discussion

### The direct association between moral identity and prosocial behavior

The findings of this study demonstrate that both internalized and symbolized moral identity are significantly and positively associated with prosocial behavior among college students. This result further supports the central role of moral identity in facilitating moral conduct. Individuals with higher levels of moral identity tend to internalize the notion of “being a moral person” as a core component of their self-concept, making them more inclined to engage in behaviors that align with social moral norms, thereby showing a greater likelihood of prosocial actions.

Notably, while both dimensions of moral identity show significant positive correlations with prosocial behavior, their underlying mechanisms differ considerably. Internalized moral identity originates more from the endorsement of an individual's intrinsic value system, emphasizing self-consistency and adherence to internal moral standards. As such, individuals with high levels of internalized identity are likely to exhibit prosocial behavior even in the absence of external validation (Wu and Yang, 2018). In contrast, symbolized moral identity is more concerned with external evaluation and the presentation of a moral image to others. Prosocial behaviors, in this case, are often motivated by the desire for social recognition and approval (Lin and Sek-yum, 2020). Therefore, prosocial behavior linked to internalized moral identity tends to be more autonomous and stable, while behavior rooted in symbolized identity may be more susceptible to external situational factors.

### The indirect association between moral identity and prosocial behavior

This study further elucidates the multiple mediation mechanisms of moral sensitivity and moral disengagement in the relationship between moral identity and prosocial behavior.

First, for internalized moral identity, both moral sensitivity and moral disengagement acted as independent mediators, and a sequential mediation pathway was also identified. Specifically, individuals with high levels of internalized moral identity are more likely to activate their moral self-schema in moral contexts, thereby enhancing moral sensitivity. At the same time, to maintain moral self-consistency, they are less inclined to adopt moral disengagement strategies. These dual pathways jointly contribute to increased prosocial behavior. However, when moral sensitivity is heightened and prosocial behavior involves substantial personal cost, individuals may adopt disengagement strategies as a self-defense mechanism to reduce inner conflict, which in turn may inhibit the actual enactment of prosocial behavior.

Second, in the case of symbolized moral identity, its indirect association with prosocial behavior was primarily realized through the independent mediation of moral sensitivity and the sequential pathway involving both moral sensitivity and moral disengagement. Given their strong concern for moral image presentation, individuals with high levels of symbolized identity also exhibit greater moral sensitivity in moral contexts. However, because their motivation is largely driven by external social feedback, such individuals may be more prone to moral disengagement—especially under cognitive overload or ego depletion—which can lead to decreased prosocial behavior (Friese and Hofmann, 2009; Hardy and Carlo, 2011).

Overall, internalized moral identity shows a more stable and direct association with prosocial behavior, whereas the association of symbolized moral identity appears more susceptible to moderation by environmental and resource-related factors.

### Why does internalized but not symbolized moral identity relate to moral disengagement?

One of the most theoretically intriguing findings of this study is that internalized moral identity was significantly negatively correlated with moral disengagement, whereas symbolized moral identity showed no significant correlation. This divergence warrants deeper interpretation.

From the perspective of self-consistency theory, individuals with high internalized moral identity view moral traits (e.g., honesty, fairness, compassion) as central to their authentic self-concept. Engaging in moral disengagement—such as justifying unethical behavior or displacing responsibility—would directly contradict their internal moral standards, creating cognitive dissonance and threatening their moral self-worth. Therefore, to preserve self-consistency, they are motivated to avoid or reject moral disengagement strategies (Bandura, 2002; Aquino et al., 2009).

In contrast, symbolized moral identity is primarily concerned with public presentation rather than internal coherence. Individuals high in symbolized moral identity care about appearing moral to others, but this external orientation does not necessarily require that their private self-evaluations align with moral standards. As a result, they may selectively employ moral disengagement when it serves impression management goals—for example, by rationalizing a self-serving behavior as unavoidable while still claiming a moral public image. Importantly, moral disengagement is often a private cognitive process that does not directly threaten one's public moral facade. Hence, symbolized moral identity shows no consistent negative association with moral disengagement; under certain conditions (e.g., when prosocial behavior is costly or when social monitoring is low), individuals high in symbolized identity might even resort to disengagement without experiencing the same level of self-condemnation as those high in internalized identity.

This interpretation aligns with prior research showing that symbolized moral identity is more context-dependent and susceptible to external incentives (Winterich et al., 2013; Hardy and Carlo, 2011). It also echoes the distinction between “moral self” as a private commitment vs. “moral self” as a social reputation. Future research could test this explanation by manipulating the salience of public vs. private self-focus and examining whether symbolized moral identity predicts moral disengagement only under conditions of low accountability or high ego depletion.

### Cultural considerations: collectivist context and its implications

It is important to interpret the above findings within the cultural context of China, a typical collectivist society. In collectivist cultures, moral identity may be shaped not only by personal values but also by strong in-group norms and relational expectations (e.g., loyalty, harmony, filial piety). Compared to individualistic Western societies, where moral identity often emphasizes personal autonomy and abstract principles, the expression of prosocial behavior in collectivist settings may be more situational and role-bound. For instance, individuals with high internalized moral identity in China may still prioritize helping in-group members over out-group strangers, a pattern that has been observed in cross-cultural studies on moral reasoning (Miller and Bersoff, 1998). Moreover, the symbolized dimension of moral identity might be particularly salient in collectivist contexts, where public face and social approval are highly valued. This could explain why symbolized moral identity in our study showed a positive correlation with prosocial behavior, albeit through different mechanisms. Future cross-cultural research should directly compare samples from individualistic and collectivist societies to examine how cultural values moderate the pathways from moral identity to prosocial behavior. Such comparisons would help determine whether the mediation roles of moral sensitivity and moral disengagement are universal or culture-specific.

### Limitations and the need for longitudinal designs

While this study provides a systematic account of how moral identity is positively associated with prosocial behavior through moral sensitivity and moral disengagement, several limitations should be acknowledged. Most critically, the study employs a cross-sectional design. Although the associations observed are consistent with our proposed theoretical model, cross-sectional data cannot support causal inferences. Therefore, terms such as “impact,” “effect,” or “pathway” used in previous sections should be understood as referring to statistical associations and theoretical directions rather than causal relationships. For example, the observed mediation sequences are theoretically plausible but could also be explained by alternative models (e.g., prosocial behavior influencing moral identity). To establish causality and understand the dynamic interplay among these variables over time, future research should employ longitudinal or experimental designs. For instance, experience sampling methods or repeated measures across multiple time points could capture how changes in moral identity precede changes in moral sensitivity, moral disengagement, and prosocial behavior.

Additionally, data were collected using self-report questionnaires. Although efforts were made to control for social desirability bias, the explicit nature of the measurement may still have introduced potential bias. Future research should consider incorporating implicit measures or experimental paradigms to improve objectivity and reliability.

Furthermore, although Harman's single-factor test indicated that common method bias was not severe, the reliance on self-report cross-sectional data means that common method variance (CMV) remains a potential limitation. Future studies should consider incorporating multi-informant reports or stronger statistical controls (e.g., unmeasured latent method construct) to further mitigate this issue. Finally, the study primarily focused on the cognitive aspects of moral sensitivity, with limited attention to the role of emotional components. Given that moral sensitivity is a multidimensional construct involving both cognition and emotion, future research should incorporate emotional variables (e.g., empathy, guilt, gratitude) to examine how cognitive-emotional interactions relate to prosocial behavior. Such exploration could contribute to the development of more scientific and personalized moral education interventions.

## Data Availability

The original contributions presented in the study are included in the article/supplementary material, further inquiries can be directed to the corresponding author.

## References

[B1] Aquino K. Freeman D. Reed A. Felps W. Lim V. K. (2009). Testing a social-cognitive model of moral behavior: the interactive influence of situations and moral identity centrality. J. Pers. Soc. Psychol. 97, 123–141. doi: 10.1037/a0015406 19586244

[B2] Bandura A. (2002). Selective moral disengagement in the exercise of moral agency. J. Moral Educ. 31, 101–119. doi: 10.1080/0305724022014322

[B3] Barr J. J. Higgins D. Alessondro A. (2007). Adolescent empathy and pro-social behavior in the multidimensional context of school culture. J. Genet. Psychol. 168, 231–250. doi: 10.3200/GNTP.168.3.231-250 18200888

[B4] Carlo G. Randall B. A. (2002). The development ofd a measure of prosocial behaviors for late adolescents. J. Youth Adolesc. 31, 31–44. doi: 10.1023/A:1014033032440

[B5] Ding X. Zhang Z. Bai J. Guo B. (2024). Translation, reliability, and validity of the Chinese version of the Moral Identity Scale in a sample of male offenders. Ethics Behav. 34, 352–368. doi: 10.1080/10508422.2023.2201944

[B6] Earp B. D. (2022). The moral repetition effect: bad deeds seem less unethical when repeatedly encountered. J. Exp. Psychol. General 151, 2562–2585. doi: 10.1037/xge0001214 35446105

[B7] Fan C. (2013). Research on Online Moral Psychology. Guangzhou: World Book Publishing Company. (in Chinese)

[B8] Fang S. Huang M. (2023). Relationship between moral elevation and prosocial behavior among college students: the mediating role of perceived social support and moderating role of moral identity. Int. J. Mental Health Promot. 25, 343–356. doi: 10.32604/ijmhp.2023.027442

[B9] Friese M. Hofmann W. (2009). Control me or I will control you: impulses, trait self-control, and the guidance of behavior. J. Res. Pers. 43, 795–805. doi: 10.1016/j.jrp.2009.07.004

[B10] Gollwitzer M. Schmitt M. Sutter R. (2005). Asymmetrical effects of justice sensitivity perspectives on prosocial and antisocial behavior. Soc. Justice Res. 18, 183–201. doi: 10.1007/s11211-005-7368-1

[B11] Hardy S. A. (2006). Identity, reasoning, and emotion: an empirical comparison of three sources of moral motivation. Motivat. Emot. 30, 205–213. doi: 10.1007/s11031-006-9034-9

[B12] Hardy S. A. Bean S. D. Olsen A. J. (2015). Moral identity and adolescent prosocial and antisocial behaviors: interactions with moral disengagement and self-regulation. J. Youth Adolesc. 44, 1542–1554. doi: 10.1007/s10964-014-0172-1 25146465

[B13] Hardy S. A. Carlo G. (2011). Moral identity: what is it, how does it develop, and is it linked to moral action? Child Dev. Perspect. 5, 212–218. doi: 10.1111/j.1750-8606.2011.00189.x

[B14] Hayes A. F. (2012). PROCESS: A versatile computational tool for observed variable mediation, moderation, and conditional process modeling [White paper].

[B15] Hertz S. G. Krettenauer T. (2016). Does moral identity effectively predict moral behavior?: a meta-analysis. Rev. General Psychol. 20, 129–140. doi: 10.1037/gpr0000062

[B16] Intishar I. N. Ampuni S. Buwono S. B. S. (2024). Academic dishonesty in online learning during the COVID-19 pandemic: the role of gender, moral self-concept, and academic self-efficacy. Jurnal Psikologi 51, 121–140. doi: 10.22146/jpsi.90823

[B17] Johnson A. J. Kim N. Thomas R. (2024). The role of organizational culture in the relationship between affective organizational commitment and unethical pro-organizational behavior. J. Manag. Psychol. 39, 845–862. doi: 10.1108/JMP-11-2022-0581

[B18] Landry A. (2022). The change of educational thought in the 21st century: the importance of moral education to college students facing the society. J. Educ. Res. Policies 4, 62–64. doi: 10.53469/jerp.2022.04(06)0.13

[B19] Lee S. Winterich K. P. Ross W. T. (2014). I'm moral, but I won't help you: the distinct roles of empathy and justice in donations. J. Consumer Res. 41, 678–696. doi: 10.1086/677226

[B20] Li Q. Hu G. (2023). Positive impacts of perceived social support on prosocial behavior: the chain mediating role of moral identity and moral sensitivity. Front. Psychol. 14:1234977. doi: 10.3389/fpsyg.2023.1234977 37908817 PMC10614638

[B21] Li Z. Wang D. Liao J. Jin Z. (2025). The relationship between moral sensitivity and prosocial behavior in college students: the mediating roles of moral disengagement and reciprocity norms. Front. Psychol. 15:1508962. doi: 10.3389/fpsyg.2024.1508962 39881686 PMC11774930

[B22] Lin W. Sek-yum S. N. (2020). The effects of anonymity, invisibility, asynchrony, and moral disengagement on cyberbullying perpetration among school-aged children in China. Child. Youth Serv. Rev. 119:105613. doi: 10.1016/j.childyouth.2020.105613

[B23] Liu C. Zhang Z. (2020). A decade of research and reflection on moral sensitivity in China. Educ. Sci. Res. 68–73. (in Chinese)

[B24] Luo X. T. Pan Y. F. (2023). Interpersonal advice interaction: decision-making, social cognition processes, and neurocomputational mechanisms (in Chinese). Chin. Sci. Bull. 68, 3809–3822. doi: 10.1360/TB-2023-0593

[B25] Lützén K. Evertzon M. (2010). Moral sensitivity in psychiatric practice. Nurs. Ethics 17, 620–631. doi: 10.1177/0969733009351951 9416106

[B26] McMahon S. D. Wernsman J. Parnes A. L. (2006). Understanding pro-social behavior: the impact of empathy and gender among African American adolescents. J. Adolescent Health 39, 135–137. doi: 10.1016/j.jadohealth.2005.10.008 16781977

[B27] Miller J. G. Bersoff D. M. (1998). The role of interpersonal attachments in moral judgment. J. Moral Educ. 27, 115–130.

[B28] Podsakoff P. M. MacKenzie S. B. Podsakoff N. P. (2012). Sources of method bias in social science research and recommendations on how to control it. Annu. Rev. Psychol. 63, 539–569. doi: 10.1146/annurev-psych-120710-100452 21838546

[B29] Reynolds S. J. (2008). Moral attentiveness: who pays attention to moral aspects of life? J. Appl. Psychol. 93, 1027–1041. doi: 10.1037/0021-9010.93.5.1027 18808223

[B30] Reynolds W. M. (1982). Development of reliable and valid short forms of the Marlowe-Crowne Social Desirability Scale. J. Clin. Psychol. 38, 119–125. doi: 10.1002/1097-4679(198201)38:1<119::AID-JCLP2270380118>3.0.CO;2-I

[B31] Schmitt M. Gollwitzer M. Maes J. Arbach D. (2005). Justice sensitivity: assessment and location in the personality space. Euro. J. Psychol. Assess. 21, 202–211. doi: 10.1027/1015-5759.21.3.202

[B32] Wang X. Yang J. Wang P. Zhang Y. Li B. Xie X. . (2019). Deviant peer affiliation and bullying perpetration in adolescents: the mediating role of moral disengagement and the moderating role of moral identity. J. Psychol. 30, 1–15. 10.1080/00223980.2019.1696733 31815608

[B33] Wang Y. (2017). The impact of moral identity on prosocial behavior under ego depletion: From the perspective of the ego depletion model (Master's thesis). Shaanxi Normal University, Xi'an, China. (in Chinese) doi: 10.1080/10508422.2023.2201944

[B34] Winterich K. P. Aquino K. Mittal V. Swartz R. (2013). When moral identity symbolization motivates pro-social behavior: the role of recognition and moral identity internalization. J. Appl. Psychol. 98, 759–770. doi: 10.1037/a0033177 23751218

[B35] Wu B. Yang Z. (2018). The impact of moral identity on consumers' green consumption tendency: the role of perceived responsibility for environmental damage. J. Environ. Psychol. 59, 74–84. doi: 10.1016/j.jenvp.2018.08.011

[B36] Wu M. Shao X. Sun X. Li N. (2017). The relationship between servant leadership, moral identity and UPB. Appl. Psychol. 23, 152–161. (in Chinese)

[B37] Yang W. Liang S. (2022). A review of Bandura's moral disengagement theory. Psychol. Res. 15, 121–125. (in Chinese)

[B38] Yang X. Wang Z. Chen H. Liu D. (2018). Cyberbullying among Chinese adolescents: the role of inter-parental conflict, moral disengagement, and moral identity. Child. Youth Serv. Rev. 86, 256–263. doi: 10.1016/j.childyouth.2018.02.003

[B39] Yang Y. Li P. Fu X. Kou Y. (2016). Orientations to happiness and subjective wellbeing in Chinese adolescents: the roles of pro-social behavior and internet addictive behavior. J. Happiness Stud. 18, 1747–1762. doi: 10.1007/s10902-016-9794-1

[B40] Zheng X. Zhu X. Zhou X. Xie F. Huang L. (2022). Internet altruistic motivation is positively associated with internet altruistic behavior: a moderated mediation model. Curr. Psychol. 42, 1–9. doi: 10.1007/s12144-022-03918-x 36373112 PMC9638391

